# Association of -1082 interleukin-10 gene polymorphism in Peruvian adults with chronic periodontitis

**DOI:** 10.4317/medoral.19823

**Published:** 2014-08-17

**Authors:** Leandro Chambrone, Amilcar Ascarza, Maria E. Guerrero, Claudio Pannuti, Manuel de la Rosa, Elmer Salinas-Prieto, Gerardo Mendoza

**Affiliations:** 1 Department of Periodontology and Oral Implantology, Dental Research Division, Guarulhos University, Guarulhos, SP, Brazil; 2 Division of Periodontics, Department of Stomatology, School of Dentistry, University of São Paulo, São Paulo, SP, Brazil; 3 Department of Periodontology, School of Dentistry, Cientifica del Sur University, Lima, Peru; 4 Department of Periodontology, University of Monterrey, Monterrey, Mexico

## Abstract

Objectives: The aim of this study was to assess association of the -1082 IL-10 gene polymorphism with chronic periodontitis CP in a Peruvian population. Study Design: Samples of venous blood and DNA were obtained from 106 Peruvian subjects: a) 53 periodontally healthy; and b) 53 with CP. The association of the -1082 IL-10 promoter sequences was assessed by Polymerase chain reaction-restriction fragment length polymorfism (PCR-RFLP). Student’s t test were used to assess the clinical parameters, as well as the χ2 test and the odds ratio (OR), with 95% confidence intervals (CI) used performed for estimates regarding genotype and allele frequencies. Results: There were statistically significant differences between groups regarding the mean bleeding on probing, mean attachment level and mean probing depth (*p* < 0.00001) indicating that the matching based on the evaluated groups was adequate. The χ2 test found a statistically significant imbalance of genotypes between groups (*p* = 0.0172). The prevalence of CP was significantly higher in subjects harboring at least one A allele at position -1082 (AA and GA genotypes) in comparison to patients with the GG genotype (OR = 2.96; CI: 0.52; 5.41; *p* = 0.0099). Equally, subjects with the AA genotype were significantly associated to a diagnosis of CP (OR = 2.71; CI: 0.38; 5.04; *p* = 0.0231). On the other hand, subjects presenting a healthy periodontal status presented at least one G allele in comparison with the AA genotype (OR = 0.37; CI: 0.05, 0.69; *p* = 0.0231). For subjects with the GG genotype, the same positive association was observed (OR = 0.34; CI: 0.06, 0.62; *p* = 0.0099). There were no significant differences between groups amongst subjects with the GA genotype (OR = 1.19; CI: 0.22, 2.16; *p* = 0.6774). Conclusions: Within the limits of this study, IL-10 gene polymorphism at position -1082 does not appear to be associated to CP. Conversely, subjects with AA genotype seem to be at an increased risk of developing CP.

** Key words:**According to MeSH documentation, chronic periodontitis, cytokines, genetic polymorphism, interleukin-10, periodontal disease.

## Introduction

Chronic periodontitis (CP) is a multifactorial disease in which the dental biofilm is the key trigger for the initiation of the inflammation process (1,2). Differently of other forms of periodontal disease, the rate of progression of the disease (i.e. periodontal tissues loss) is slow to moderate, and it is commonly associated with the presence of the aetiological agent (3,4).

It has been demonstrated that biofilm-induced inflammation leads to a sequence of innate and modified immune host responses, that stimulates the production of cytokines and chemokines, and that may be modulated by genetic factors (5,6). Among these inflammatory mediators, interleukin-10 (IL-10) has been considered and important contributor to the pathogenesis of periodontal diseases (5-7). IL-10 is a fundamental cytokine involved in the infection’s regulatory processes (for some T-cell subgroups), as well as it precludes for autoimmunity because of the tolerance to self-antigens (5). As a result, it plays a role on: 1) suppressing macrophages and other important cells; 2) controlling the inflammation; and 3) preventing tissue breakdown (5,7-10).

With respect to the role of genetic predisposition (GP), a couple of studies performed on twin brothers found that GP can increase the risk of CP (11,12), as well as GP has been linked to the capacity of producing IL-10 (13,14). Located on chromosome 1q31-q32, IL-10 gene presents diverse discrepancies in the promoter region that leads to dissimilarities on the production of the related-anti-inflammatory citokine, and to consequent modifications of the inflammatory processes (13,14). Together with the plausibility of genetic polymorphisms (GP) of IL-10 influencing different conditions of the body, it has been reported the possible influence of gene expression in regulating CP (15-18). On the other hand, additional studies failed to support the association of GP and periodontitis (19,20).

Another study on the prevalence of interleukin-1 periodontal genotype in a Hispanic dental population, Caffesse *et al*. found a genotype positive prevalence of 26% (21). This same group studied the effect of interleukin-1 GP in a periodontally healthy Hispanic population treated with mucogingival surgery. In this study 22 subjects who were treated for class I and class II recession-type defects were evaluated for the response to mucogingival surgery on a population with a 26% genotype positive prevalence. Results indicated that 5 out of 22 subjects were genotype positive. Treatment of the localized recessions provided similar amount of coverage in genotype positive and negative subjects. They concluded that periodontal health can be maintained with proper preventive maintenance irrespective of the genotype present (22).

Despite of these surveys conducted in different populations, there have been few studies focusing on the influence of IL-10 GP among Latin-American populations, and none on Peruvian (Amerindian-Spanish) subjects, in terms of CP regulation. Thus, the purpose of this study was to assess association of the -1082 IL10 GP with CP.

## Material and Methods

- Study design and population

One hundred and six systemically healthy, non-smoking subjects, 40 male and 66 female, 20 to 74 years old (mean age: 43.4 years), participated in this cross-sectional study. These subjects were selected from an initial sample of 800 patients who were referred for dental treatment at the dental clinic of Científica del Sur University between July 2012 and March 2013. The protocol of the study was prepared according to The Strengthening the Reporting of Observational Studies in Epidemiology (STROBE) statement (23). It was approved by the Ethics on Research Committee of Faculty of Dentistry, Científica del Sur University, and was conducted in accordance with the Helsinki Declaration of 1964 as revised in 2008. The subjects participating in the study were volunteers who received detailed information regarding the proposed research and provided signed consent.

- Inclusion and exclusion criteria

The subjects were enrolled in the study when the following inclusion criteria were met: 1) age > 20 years; 2) at least 15 teeth, excluding third molars; and 3) a non-smoking status. All consecutive patients who met these inclusion criteria were invited to take part in the study. Patients with a diagnosis of aggressive periodontitis, a known systemic disease (e.g., acquired immunodeficiency syndrome [AIDS], diabetes mellitus, blood disease problem), pregnant/lactate, who were submitted to periodontal therapy in the previous 12 months, or antimicrobial, anti-inflammatory and immunosuppressive therapies during the previous 6 months were not included in the study.

- Sample size calculation 

Sample size was calculated considering a two-sided significance level of 5%, a power of 80%, the proportion of subjects with CP in the non-exposed group of 38% and proportion of subjects with CP in the exposed group (i.e. AA -1087 Genotype) of 69%, based on a recent study (7). Considering the Fleiss method with continuity correction factor, a sample size of 47 per group would be necessary.

- Clinical examination and experimental groups

Full medical and dental histories were obtained. Data included full mouth probing depth (PD) measured at six sites around teeth, clinical attachment level (CAL) and bleeding on probing (presence or absence) using a UNC-15 periodontal probe recorded by two trained and calibrated periodontists (GM and LP) (intraclass correlation coefficient > 0.82 for PD and CAL). Patients without a history of periodontitis and no sites with PD and CAL > 3 mm concomitantly were included in the periodontally healthy group (Group H; n=53) and those exhibiting bleeding on probing and at least four teeth with a probing depth (PD) >4 mm were entered to the chronic periodontitis group (Group CP; n=53). Chronic periodontitis was defined according to the criteria established by the American Academy of Periodontology (1,3).

- Molecular Blood Analysis (Linfocyte Isolation and DNA Extraction)

Blood was centrifuged at 7000 rpm x 5min for the molecular analysis, separating the plasma and recuperating the leucocytes, and after that 3 ml of hypotonic solution TE 20:5 was added. This process was repeated until the precipitation could get a white colour, which was then embedded in 200ul of TE 20:5. Once obtained, it was freezed at -20ºC and then thawed at 54ºC. Afterwards 22.5ul of a SDS 10% solution was added with a final concentration of 1.13%, as well as 5ul of proteinase K at 500ug/ml was aggregated. It was then mixed and incubated at 54ºC, for an entire night. At a latter point 500 ul of chloroform solution of isopropilic alcohol 24:1 Ice was added, agitated for a 10min period. After that this solution of centrifuged at 5rpm for 10 min, recuperating the supernatant on other container with precautions for not touching the interphase. At this time, 174ul of Ammonium Acetate with a final concentration of 7M. After the mix a floating fluff was obtained. It was then centrifuged at 14000 rpm x 3 opted and then 1ml of a 70% solution of ethanol was added, so as to eliminate the remaining proteins and the ammonium acetate. A buffer TE 20:1 of 50 to 500 ul was added. In order to preserve the DNA sample, it was placed on 4ºC for 1 week, before it was stored at -20ºC.

- Polymerase chain reaction-restriction fragment length polymorfism (PCR-RFLP)

Samples were genotyped by PCR-RFLP method for IL-10 -1082. According to the procedures reported by Brett *et al*. (24), PCR was performed in a final volume of 50ul containing 100ng Genomic DNA, 20mM Tris-Hcl (pH - 8.4), 2mM of MgCL2, 0.2 mM of every deoxynucleotide (dNTP) (New England Labs Inc., Ipswich, MA, USA) and 0.8 uM of every primer (5′-TCT GAA GAA GTC CTG ATG TCA CTG-3′ y 5′- ACT TTC ATC TTA CCT ATC CCT ACT TCC-3′ - (Fermentas, Thermo Fisher Scientific Inc, Waltham, MA, USA), and 1.5 U of Taq DNA polymerase. Then, the amplification protocol was conducted as follows : 1) Pre-PCR (95°C for 4 minutes); 2) PCR (40 cicles: 95°C for 1 minute, 55°C for 1 minute, and 72°C for 1 minute); and 3) elongation (72°C for 1 minute, 4°C for indefinite). After these previous procedures, DNA was digested with restriction fragments and separated by size using electrophoresis. RFLP amplification protocols were directed with the restriction enzyme Mnll, as well as the fragments was visualized on an agarose gel at 3%. The expect results were: 1) allele G: 28bp/58bp/110; and 2) allele A: 58bp/138bp.

- Statistical analysis

Descriptive statistics were used to synthetize collected data using the subject as the unit of analysis. Statistical analysis of the clinical parameters was carried out to compare the differences between groups using the Two Sample t-test. Differences between groups in terms of the distribution of frequencies of genotypes and allele carriage (G or A) in healthy (H) and chronic periodontitis (CP) subjects were assessed by the Chi-Square (χ2) test. Additionally, the chance associated with individual or combined genotypes was calculated as the odds ratio (OR), with 95% confidence intervals (CI). A significance level for rejection of the null hypotheses was set at α= 0.05. The analyses were performed using a software package (NCSS 2007 (Number Cruncher Statistical System, NCSS, Kaysville, UT, USA).

## Results

- Subject characteristics

Differences in clinical measurement between subjects with or without CP are depicted in  Table 1. There were statistically significant differences between H and CP groups for the mean BoP, mean CAL and mean PD (*p* < 0.00001). These differences showed that the matching based on these two groups was adequate.

**Table 1:**
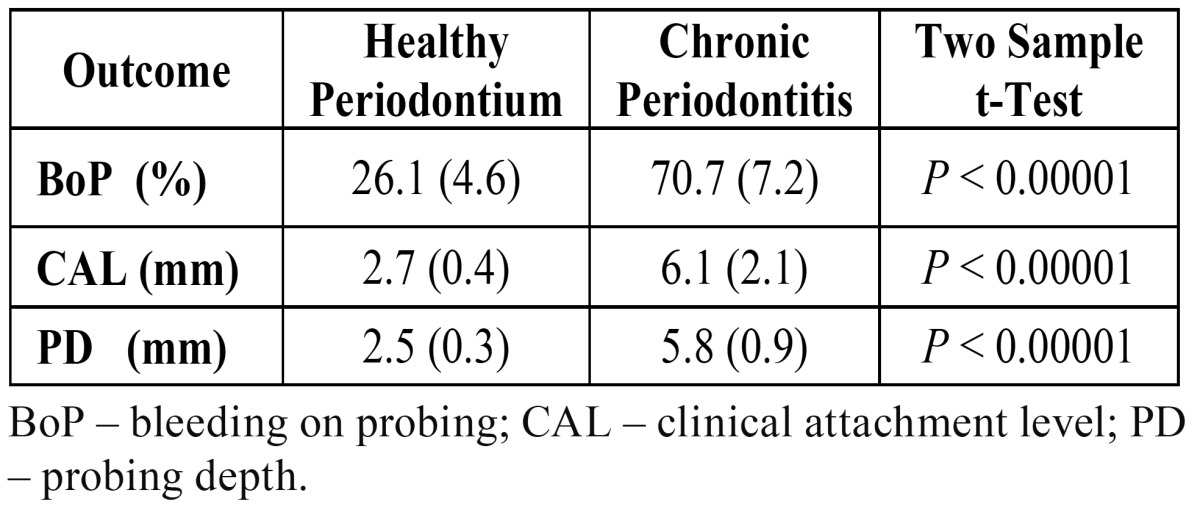
Clinical measurements: mean and standard deviation (SD).

- Genotype and allele frequencies

The distribution of frequencies of genotypes and allele carriage of the IL-10 -1082 in subjects with or without CP is described in  Table 2. The Chi-Square test found a statistically significant imbalance of genotypes between the groups of periodontally healthy patients and those with CP (*p* = 0.0172). With respect to the association between CP, genotype and allele frequency, the ORs were estimated as following: 1) when OR>1, the genotype or the presence of at least one specific allele favored the development of CP; 2) when OR <1, the genotype or the presence of at least one specific allele favored the maintenance of a healthy periodontium. The prevalence of CP was significantly different between carriers of the AA and GG genotypes at position -1082 A/G in comparison to GA genotype. CP was significantly higher in subjects harboring at least one A allele at position -1082 A/G (AA and GA genotypes) in comparison to patients with the GG genotype (OR = 2.96; CI: 0.52; 5.41; *p* = 0.0099). Equally, subjects with the AA genotype were significantly linked to a diagnosis of CP (OR = 2.71; CI: 0.38; 5.04; *p* = 0.0231).

**Table 2:**
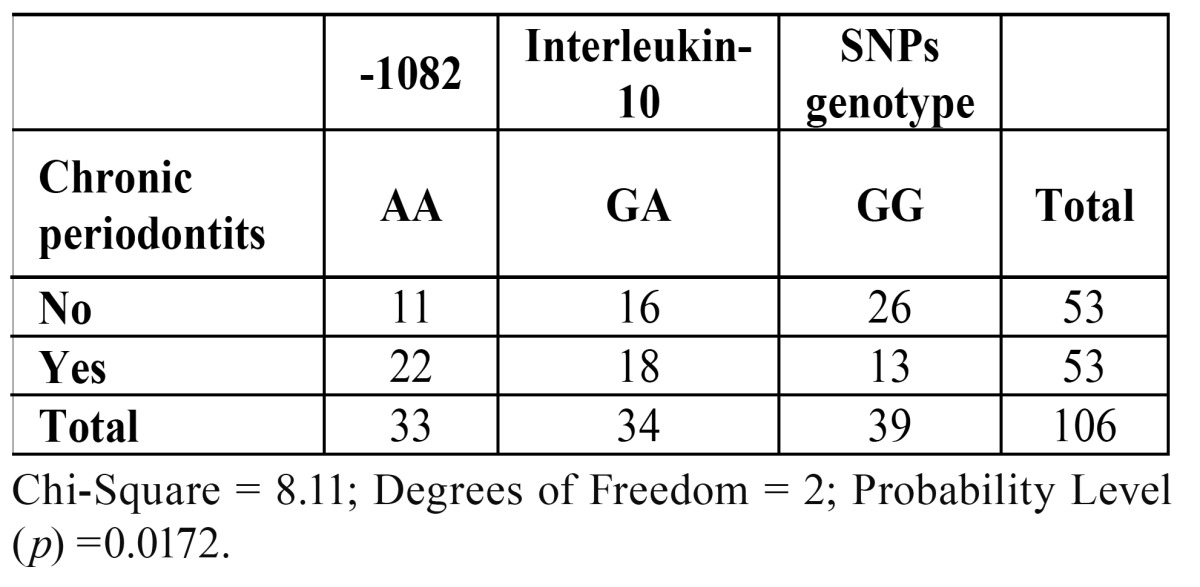
Distribution of subjects according to -1082 interleukin-10 gene polymorphism.

On the other hand, subjects presenting a healthy periodontal status presented at least one G allele (GG or GA genotypes) in comparison with the AA genotype (OR = 0.37; CI: 0.05, 0.69; *p* = 0.0231). For subjects with the GG genotype, the same positive association was observed (OR = 0.34; CI: 0.06, 0.62; *p* = 0.0099). In addition, there were no significant differences between groups amongst subjects with the GA genotype (OR = 1.19; CI: 0.22, 2.16; *p* = 0.6774).

## Discussion

This study assessed the association between CP and the polymorphisms of the IL-10 promoter and in a Peruvian (Amerindian-Spanish) population. According to the results found by the statistical analyses applied, the significant differences in clinical results showed that the selection of patients with or without CP was correctly performed. Regarding the distribution of frequencies of genotypes and allele carriage of the IL-10 -1082, these were not balanced between groups (*p* = 0.0172) and the following assumptions could be addressed: 1) subjects harboring at least one A allele seems to be more susceptible to CP when compared to subjects with the GG genotype; and 2) subjects harboring at least one G allele seems to be less susceptible to CP when compared to subjects with the AA genotype.

Moreover, differences between groups were not found for subjects with the GA genotype (*P*=0.6774). These outcomes are in line with data from other populations that evaluated the frequency of -1087 IL-10 gene polymorphism between subjects with or without CP (16,24-26). Conversely, results from Chinese, Jordanian and Swedish did not show the same. For instance, Loo *et al*. (27) found that Chinese subjects with CP presented a significant lower frequency of a -1082 IL-10 AA genotype than those without CP (*p* < 0.001), with odds ratio of 6.5 for subject harboring at least one G allele (GG and GA genotypes) when compared with AA genotype amongst subject with CP. As a result, these authors suggested that the prevalence of subjects with a GG genotype is significantly lower in subjects without CP than in subjects with CP (28). Moreover, Berglundh *et al*. (15) reported similar results. They found that subjects with CP were positively associated to a GG genotype than subjects without CP (OR = 6.1). Indeed, it has been suggested that such contrasting outcomes could be associated to genotypic differences in cytokine genes between ethnically different populations (16). Furthermore, other environmental condition, such as smoking might explains contradictory outcomes (7,24).

In addition, it has been suggested that the outcomes of previous studies that found positive associations between periodontal disease and GP might be produced by type 1 errors. Schaefer *et al*. (28) performed a large-scale, replication study in which potential candidate genes of periodontitis were assessed, and no associations were identified. These authors also suggested that the lack of associations could be related to ethnical homogeneity of the studied populations. In this short communication an Amerindian-Spanish population was assessed for the first time, and it might be possible that a type I error may have occurred. Sample size calculation has been performed to avoid such a type of statistical error, but it might not has been enough to assess the real link of GP and CP; however, it might be argued that some genetic heterogeneity between distinct populations may allow that high-risk variants in the human genome might be present, and thus real associations may be present. As a result, these are important aspects for the present preliminary study and require careful interpretation.

Within the limits of this study, the association of -1082 interleukin-10 gene polymorphism with CP was assessed for the first time in a Peruvian population. Based on the present results, it can be concluded that IL-10 polymorphisms is not associated to CP. Conversely, subject with AA genotype seems to be an increased risk of developing CP.

## References

[1] Armitage GC (1999). Development of a classification system for periodontal diseases and conditions. Ann Periodontol.

[2] Schätzle M, Löe H, Lang NP, Heitz-Mayfield LJA, Bürgin W, Ånerud Å (2003). Clinical course of chronic periodontitis. J Clin Periodontol.

[3] Armitage GC (2004). Periodontal diagnoses and classification of periodontal diseases. Periodontol 2000.

[4] Chambrone L, Chambrone D, Lima LA, Chambrone LA (2010). Predictors of tooth loss during long-term periodontal maintenance: a systematic review of observational studies. J Clin Periodontol.

[5] Preshaw PM, Taylor JJ (2011). How has research into cytokine interactions and their role in driving immune responses impacted our understanding of periodontitis?. J Clin Periodontol.

[6] Dentino A, Lee S, Mailhot J, Hefti AF (2013). Principles of periodontology. Periodontol 2000.

[7] Jaradat SM, Ababneh KT, Jaradat SA, Abbadi MS, Taha AH, Karasneh JA (2012). Association of interleukin-10 gene promoter polymorphisms with chronic and aggressive periodontitis. Oral Diseases.

[8] Couper KN, Blount DG, Riley EM (2008). IL-10: the master regulator of immunity to infection. J Immunol.

[9] O'Garra A, Barrat FJ, Castro AG, Vicari A, Hawrylowicz C (2010). Strategies for use of IL-10 or its antagonists in human disease. Immunol Rev.

[10] Ouyang W, Rutz S, Crellin NK, Valdez PA, Hymowitz SG (2011). Regulation and functions of the IL-10 family of cytokines in inflammation and disease. Ann Rev Immunol.

[11] Michalowicz BS, Aeppli D, Virag JG, Klump DG, Hinrichs JE, Segal NL (1991). Periodontal findings in adult twins. J Periodontol.

[12] Michalowicz BS, Diehl SR, Gunsolley JC, Sparks BS, Brooks CN, Koertge TE (2000). Evidence of a substantial genetic basis for risk of adult periodontitis. J Periodontol.

[13] Jin X, Hu Z, Kang Y, Liu C, Zhou Y, Wu X (2012). Association of IL-10-1082 G/G genotype with lower mortality of acute respiratory distress syndrome in a Chinese population. Mol Biol Rep.

[14] Zhang G, Manaca MN, McNamara-Smith M, Mayor A, Nhabomb AN, Berthoud TK (2012). Interleukin-10 (IL-10) Polymorphisms Are Associated with IL-10 Production and Clinical Malaria in Young Children. Infection and Immunity.

[15] Berglundh T, Donati M, Hahn-Zoric M, Hanson LA, Padyukov L (2003). Association of the-1087 IL 10 gene polymorphism with severe chronic periodontitis in Swedish Caucasians. J Clin Periodontol.

[16] Scarel-Caminaga RM, Trevilatto PC, Souza AP, Brito RB, Camargo LE, Line SR (2004). Interleukin 10 gene promoter polymorphisms are associated with chronic periodontitis. J Clin Periodontol.

[17] Sumer AP, Kara N, Keles GC, Gunes S, Koprulu H, Bagci H (2007). Association of interleukin-10 gene polymorphisms with severe generalized chronic periodontitis. J Periodontol.

[18] Cullinan MP, Westerman B, Hamlet SM, Palmer JE, Faddy MJ, Seymour GJ (2008). Progression of periodontal disease and interleukin-10 gene polymorphism. J Periodontal Res.

[19] Kinane DF, Hodge P, Ellis R, Gallagher G (1999). Analysis of genetic polymorphisms at the interleukin-10 and tumour necrosis factor loci in early-onset periodontitis. J Periodont Res.

[20] Hennig BJ, Parkhill JM, Chapple IL, Heasman PA, Taylor JJ (2000). Dinucleotide repeat polymorphism in the interleukin-10 gene promoter (IL-10.G) and genetic susceptibility to early-onset periodontal disease. Genes Immun.

[21] Caffesse R, De la Rosa RM, De la Rosa GM, Mota L (2002). Prevalence of Interleukin 1 periodontal genotype in a Hispanic dental population. Quintessence Int.

[22] Caffesse RG, De La Rosa RM, De La Rosa GM, Weltman R (2002). Effect of interleukin-1 gene polymorphism in a periodontally healthy Hispanic population treated with mucogingival surgery. J Clin Periodontol.

[23] von Elm E, Altman DG, Egger M, Pocock SJ, Gøtzsche PC, Vandenbroucke JP (2008). The Strengthening the Reporting of Observational Studies in Epidemiology (STROBE) statement: guidelines for reporting observational studies. J Clin Epidemiol.

[24] Brett PM, Zygogianni P, Griffiths GS, Tomaz M, Parkar M, D'Aiuto F (2005). Functional gene polymorphisms in aggressive and chronic periodontitis. J Dent Res.

[25] Yamazaki K, Tabeta K, Nakajima T, Ohsawa Y, Ueki K, Itoh H (2001). Interleukin-10 gene promoter polymorphism in Japanese patients with adult and early-onset periodontitis. J Clin Periodontol.

[26] Reichert S, Machulla HK, Klapproth J, Zimmermann U, Reichert Y, Gläser CH (2008). The interleukin-10 promoter haplotype ATA is a putative risk factor for aggressive periodontitis. J Periodont Res.

[27] Loo WTY, Fan CB, Bai LJ, Yue Y, Dou YD, Wang M (2012). Gene polymorphism and protein of human pro-and anti-inflammatory cytokines in Chinese healthy subjects and chronic periodontitis patients. Journal of Translational Medicine.

[28] Schaefer AS, Bochenek G, Manke T, Nothnagel M, Graetz C, Thien A (2013). Validation of reported genetic risk factors for periodontitis in a large-scale replication study. J Clin Periodontol.

